# Efficacy of Single-Dose Albendazole and Albendazole Plus Ivermectin for Soil-Transmitted Helminth Infection in Children in the Peruvian Amazon

**DOI:** 10.4269/ajtmh.23-0497

**Published:** 2024-05-28

**Authors:** Greisi Curico, Paul F. Garcia Bardales, Tackeshy N. Pinedo Vasquez, Wagner Valentino Shapiama Lopez, Maribel Paredes Olortegui, Francesca Schiaffino, Pablo Peñataro Yori, Josh M. Colston, Thomas G. Flynn, Graciela Meza Sánchez, Hermann Silva Delgado, Richard A. Oberhelman, Margaret N. Kosek

**Affiliations:** ^1^ Asociación Benéfica PRISMA, Iquitos, Peru;; ^2^ Faculty of Veterinary Medicine, Universidad Peruana Cayetano Heredia, Lima, Peru;; ^3^ Division of Infectious Disease and International Health, University of Virginia, Charlottesville, Virginia;; ^4^ Universidad Nacional de la Amazonia Peruana, Iquitos, Peru;; ^5^ Tulane University, New Orleans, Louisiana

## Abstract

In countries where soil-transmitted helminth (STH) infections are endemic, deworming programs are recommended to reduce morbidity; however, increasing levels of resistance to benzimidazoles are of concern. In an observational study in Peru, we studied the clinical efficacy of 400 mg of albendazole 20 days after treatment among children aged 2–11 years. Of 426 participants who provided samples, 52.3% were infected with a STH, 144 (33.8%) were positive for *Ascaris* (41.8% light, 50.8% moderate, and 7.4% heavy infections), 147 (34.5%) were positive for *Trichuris* (75.2% light, 22.5% moderate, and 2.3% heavy infections), and 1.1% were positive for hookworm species (100% light infections). Additional stool samples were examined at 20, 90, and 130 days after the initial treatment. At 20 days post-administration of albendazole, the cure rate (CR) of *Ascaris* infection was 80.1% (95% CI: 73.5–86.7), and the egg reduction rate (ERR) was 70.8% (95% CI: 57.8–88.7); the CR for *Trichuris* infection was 27.1% (95% CI: 20.0–34.3), and the ERR was 29.8% (95% CI: –1.40 to 57.5). Among participants with persistent or recurrent infections with *Trichuris*, the combined therapy of albendazole (400 mg) and ivermectin at 600 *µ*g/dose increased overall CR for *Trichuris* infection to 75.2% (95% CI: 67.3–83.2%) with an ERR of 84.2% (95% CI: 61.3–93.8%). Albendazole administration alone for the control of STH was associated with high rates of treatment failure, especially for *Trichuris*. Combined single doses of albendazole and ivermectin was observed to have improved efficacy.

## INTRODUCTION

Soil-transmitted helminth (STH) infections are distributed in tropical and subtropical areas, such as sub-Saharan Africa, the Americas, China, and South Asia, where approximately 1.5 billion people are estimated to be infected.^1^ More than 267 million preschool children (1–4 years) and 568 million school-age children (5–14 years) living in areas at high risk of transmission are recommended to receive periodic deworming against these infections.^2^^–^^4^

The programmatic administration of anthelmintic drugs recommended by the WHO contributes to a reduction in transmission in STH.^5^ Deworming by preventive chemotherapy established by WHO and PAHO to eliminate STH morbidity in areas with prevalence >50% is based on a population based treatment (albendazole 400 mg or mebendazole 500 mg) twice a year. In areas with prevalence >20% and <50%, once-yearly treatment is recommended.^6^^,^^7^ In Peru, population-level deworming twice a year in primary health care centers and schools takes place through a program first established in 2017 and reiterated in 2021 guidelines.^8^ There is, however, growing concern that despite ease of use, the current strategy, which uses a single dose of a single agent, the efficacy of this approach is limited—in particular, in the control of *Trichuris* infection.^9^^–^^13^

We conducted a prospective observational study designed to evaluate the programmatic efficacy of current STH guidelines. Specifically, we sought to evaluate the clinical response to albendazole monotherapy and time to reinfection of STH among children in the Peruvian Amazon who receive preventive treatment every 6 months according to the Peruvian Ministry of Health guidelines. A secondary aim was to measure the efficacy of combined therapy of albendazole with high-dose ivermectin in the treatment of children who remained persistently infected with *Trichuris* or other STH after treatment with albendazole monotherapy.

## MATERIALS AND METHODS

### Study area and design.

A prospective cohort study was conducted in the city of Iquitos, Loreto, Peru. Children aged 2–11 years were enrolled, each with an individual duration of participation of 4.5 months with four study visits at specific times between October 2021 and January 2023.

The study was conducted in the jurisdiction of the 6 de Octubre Health Center, which includes riverine, peri-urban, and urban environments. The riparian zone is located on the opposite bank of the Itaya River from the city of Iquitos. It is prone to flooding and lacks basic services, such as electricity and potable water. The peri-urban area is adjacent to the city and extends down to the river, with houses on stilts or houseboats that float on the surface of the river. Many of them have electricity and potable water and are usually close to the health center.

A community census was conducted beforehand to identify eligible children. In the riverine area, all children who met the inclusion criteria were invited to enroll. In the peri-urban and urban area, where there are defined maps with streets and addresses, we recruited a randomized sample of approximately 30% of the eligible target population through approaching every third house and closing blocks clockwise. Demographic data were collected, and children’s weight and height were recorded at the beginning and end of follow-up for each participant. Inclusion criteria included the absence of deworming treatments in the 3 months before enrollment, as well as permanent residence in the study area with no plans to move over the following 6 months. A detailed study protocol has been published previously.^14^

History of time since last deworming was obtained. Children had height measured with a commercially available measuring board, and weight was measured to the nearest 10 g with a Tanita scale. The WHO standards were used to express measures as height-for-age Z-scores, weight-for-age Z-scores, and weight for height Z-scores using the 2006 growth standards for children under 60 months of age and the 2007 standard for children 5–11 years of age. Because interpretation of nutritional status is age dependent, the results are presented separately for these different age categories.

### Sample processing.

A 100-mL sterile plastic vial with a cap was provided to all participants for stool collection 1 day before each visit (first pretreatment sample as well as second, third, and fourth samples on study days 20, 90, and 130 relatives to the day of the first administration of albendazole to measure treatment efficacy and reinfection at 11%, 50%, and 75% of the recommended 6-month interval). These stool samples were analyzed using direct fecal smear and the Kato–Katz technique using a 41.7-mg template.^15^ All Kato–Katz smears were evaluated independently by two microscopists, and the mean estimate used to enumerate the egg count for each species separately.

### Treatment and clinical follow-up.

**Figure 1.**
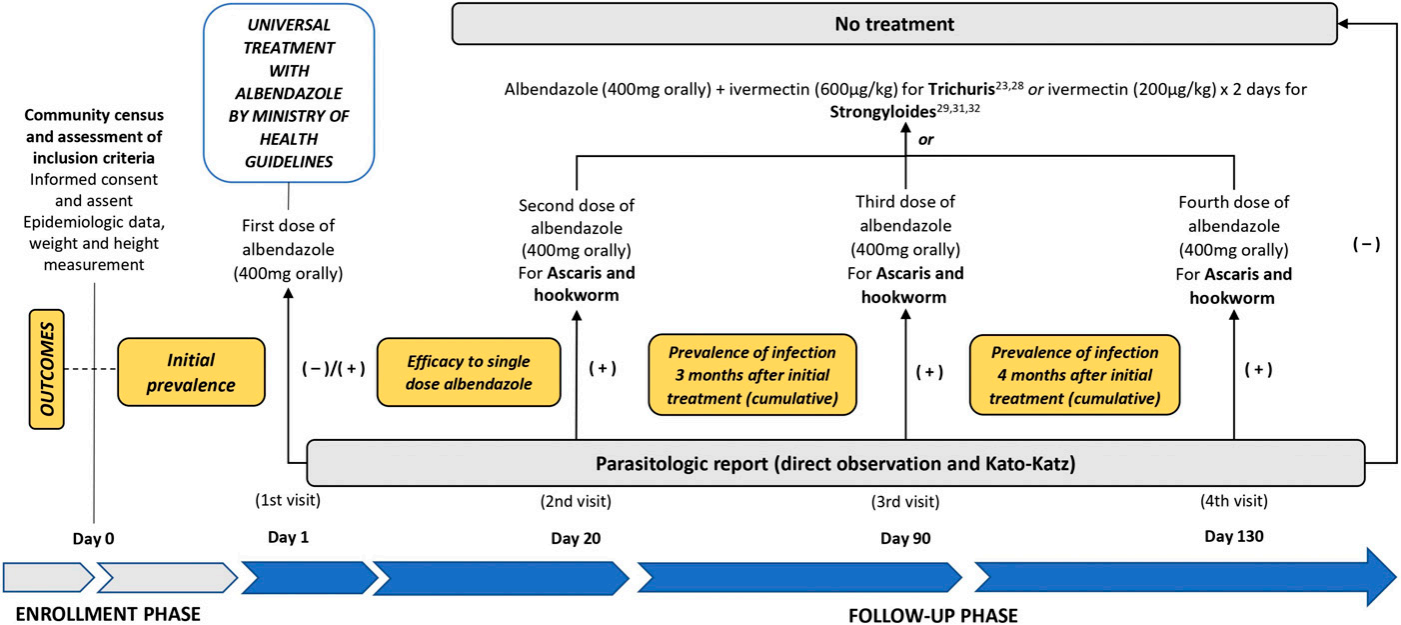
Diagram of study procedures and primary outcomes. All subjects received an observed oral 400-mg dose of albenzadole per Peruvian National Guidelines on study day 1 independent of results of stool examination. Subjects had additional stools collected on study days 20, 90, and 130. If subjects remained negative by Kato–Katz and direct observation on follow-up exams, they received no additional antiparasitic therapy. If they were positive on samples from days 20, 90, or 130, they received additional parasite-directed therapy. Individuals with hookworm or *Ascaris* received an additional dose of albendazole 400 mg. For individuals with *Trichuris*, because of extensive data showing low treatment efficacy of albendazole alone, children were given a single combined dose of ivermectin at a dose of *200 µg*/kg in addition to albendazole 400 mg. Children with *Strongyloides* received 2 days of oral therapy with ivermectin dosed at *200 µg*/kg.

Once the initial stool sample was collected, albendazole 400 mg was administered orally according to the guidelines of the Peruvian Ministry of Health (RM No. 479-2017-MINSA) as part of the universal treatment policy.^8^ Treatment was directly observed by a study team member. When parasites were found in the samples after the initial treatment, individualized treatment was administered according to current treatment guidelines with preference for effective regimens that were administered in a single dose for programmatic purposes. For *Ascaris lumbricoides* and hookworm, albendazole 400 mg was administered as a single oral dose. For *Trichuris trichiura*, both ivermectin 600 *µ*g/kg with albendazole 400 mg were administered together,^11^^,^^16^ and for *Strongyloides stercolaralis*, ivermectin 200 *µ*g/kg/day for 2 days was administered^10^^,^^17^^,^^18^ (see Figure 1).

### Data management.

Data was entered using a double data entry system in an ACCESS database management system (Microsoft, Seattle, WA). Double data entry was performed and differences resolved by a supervisor.

### Ethical review.

The project was reviewed and approved by the Institutional Ethics Committee of the Asociación Benéfica PRISMA and the Biomedical Institutional Review Board of Tulane University.

## STATISTICAL ANALYSES

### Definitions.

A child was designated as infected with a specified parasite if diagnostic forms were present on either microscopy of direct fecal smears or by Kato–Katz. Infections were categorized as light, moderate, or heavy per WHO guidelines^19^^,^^20^ for each parasite: *A*. *lumbricoides*—light (1–4,999 eggs per gram [epg]), moderate (5,000–49,999 epg), and heavy (>50,000 epg); *T. trichiura*—light 999 epg), moderate (1,000–9,999 epg), and heavy (>10,000 epg); hookworm—light infection (1–1,999 epg), moderate (2,000–3,999 epg), and heavy (>4,000 epg).^21^

Treatment efficacy was determined through the calculation of cure rates (CRs) and egg rate reduction rates (ERRs). Individuals were considered cured if they converted from positive to negative for a particular STH infection after an observed treatment was administered. The CRs were calculated by dividing the number of individuals positive at the preintervention survey who were negative at the relevant post-intervention survey by the number of individuals who were positive at the preintervention survey. Changes were evaluated for statistical significance with the Yates χ^2^ test. Cure rates were calculated separately and specified as Cure20 (cure after baseline examination and treatment with 400 mg of albendazole as measured at day 20), Cure90 (cure after clinically directed therapy on day 20), and Cure130 (cure after clinically directed therapy on day 90). Cure rates were calculated based on numbers of individuals who supplied stools that enabled cure status to be determined at each treatment cycle. Efficacy of medication in decreasing the intensity of STH was measured for each pathogen after each treatment cycle.

The CR was calculated as follows:

$$\text{CR}\;\left(\text{\%}\right)=100*\left(\begin{array}{c} \frac{\;\#\;\text{cured}\;\text{post-treatment}\;}{\text{total}\;\#\;\text{of}\;\text{pre-treatment}\;\text{positives}} \end{array}\right)$$

The ERR was calculated as follows:

$$\text{ERR}\;\left(\text{\%}\right)=100*\left(\begin{array}{c} 1-\frac{\text{arithmetic}\;\text{mean}\;\text{egg}\;\text{counts}\;\text{at}\;\text{follow-up}}{\text{arithmetic}\;\text{mean}\;\text{at}\;\text{baseline}} \end{array}\right),$$

per WHO norms.^22^^,^^23^ Treatment efficacy of medication was measured separately for each pathogen at each treatment cycle. Confidence intervals were calculated using the bootstrap method with 9,999 iterations. Assessments of treatment efficacy were calculated only for STH with a baseline prevalence that exceeded 10%.

Data analysis was performed in R (version 4.3.1). Sankey diagrams were generated using the networkD3 package (version 0.4).

Reinfections were defined as a reversion to test positivity in an individual who had previously been cured after antihelminthic therapy. Rates of reinfection for each parasite are reported separately for days 90 and 130 and inclusive of all participating individuals who had a stool sample available for analysis at the most distant time point relevant to the period being reported.

## RESULTS

### Screening and enrollment.

**Figure 2.**
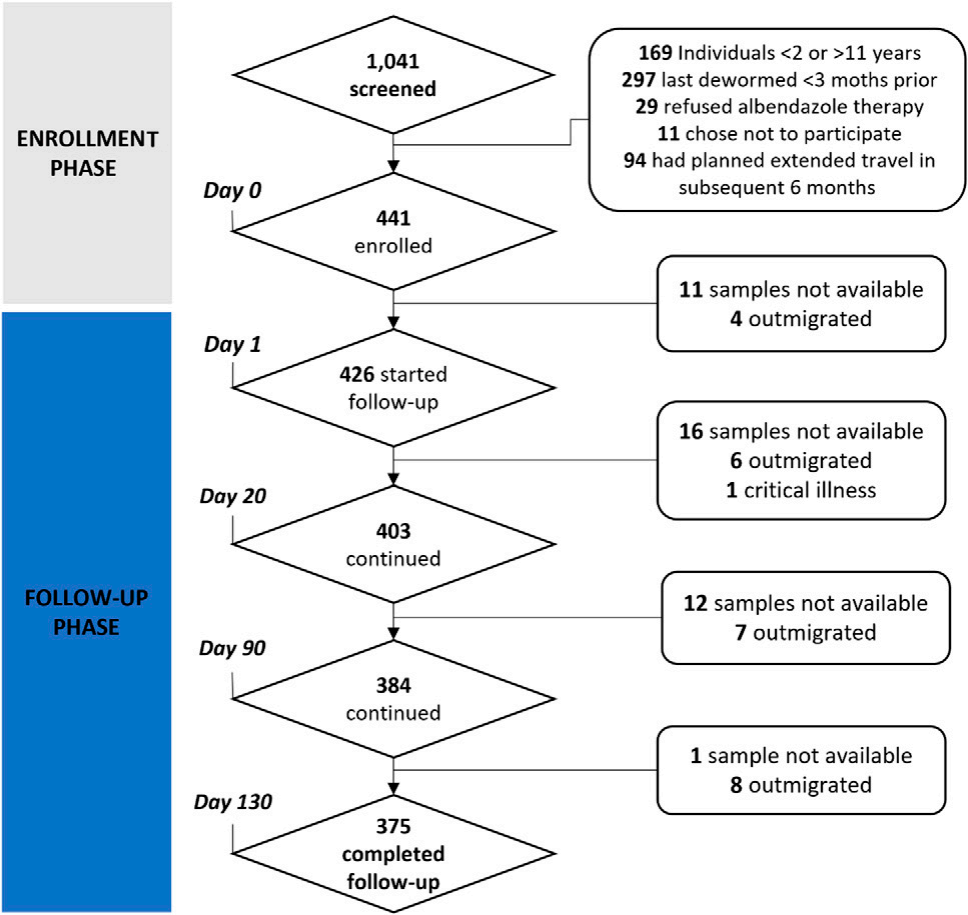
Diagram of recruitment of participants.

One thousand and forty-one children were screened to participate in the study, of whom 600 (57.6%) were excluded (297 children who were dewormed within the previous 3 months, 169 children were under 2 or over 11 years of age, 94 children would be moving within 6 months, 11 children’s parents did not wish to have their child participate, and 29 declined deworming (see Figure 2). Retention in the study was high, with 91.4% (403/441) of enrolled participants providing the first post-treatment stool at day 20, 87.1% (384/441) providing both the first (D20) and second post-treatment stool (D90), and 85% (375/441) of enrollees provided the four specified stool samples during the 4.5-month study period.

### Sociodemographic characteristics of study population.

Females made up 51.6% of the study population with a mean age of 6.45 years (see Table 1). The majority of participants lived in an urban or peri-urban area (64.2%), and 35.8% of the population lived in a rural riverine zone. Approximately one-third (32.0%) had a family size of fewer than five, 65.9% had a family size of between five and nine, and 2.3% had a family size of more than nine individuals. Improved water source (generally piped and treated water) was reported by 76.9% of participants, where river water was the principal source of water for 19.0% of the study population. Nearly half (46.2%) of the children enrolled in the study had been dewormed in the previous 3–6 months, demonstrating adequate coverage of the current deworming program in the area; as 297 of children were excluded for having been dewormed in the previous 3 months for an estimated program coverage of 67.9% in the previous 6 months. An additional 25.6% of children were reported to have been dewormed in the previous 7–12 months for at least yearly coverage in 93.4% of the population under study. Chronic malnutrition was common, and 26.9% of children under age 60 months were stunted (HAZ < –2).

**Table 1:** Demographic characteristics of the subjects at enrollment

Baseline Characteristics	% (*n/N*)
Sex	
Male	48.4% (212/441)
Female	51.6% (229/441)
Mean age (years)	6.46 (SD 2.84)
Residence area	
Urban	36.5% (161/441)
Peri-urban	27.7% (122/441)
Riverine	35.8% (158/441)
Family size	
<5	32% (141/441)
5–9	65.7% (290/441)
>9	2.3% (10/441)
Last anthelmintic treatment*	
>3–6 months	46.2% (204/441)
7–12 months	26.1% (115/441)
>12–72 months	27.7% (122/441)
Water availability	
Improved water	76.9% (339/441)
River water	19% (84/441)
Well water	3.4% (15/441)
Other	0.7% (3/441)
Nutritional status (<5 years)	(134/441)
WAZ	Mean: –1.05, SD: 1.30
Stunting (HAZ ≤ –2)	26.9% (36/134)
Height-for-age (HAZ)	Mean: –1.41, SD: 1.27
Underweight (WAZ ≤ –2)	23.1% (31/134)
WHZ	Mean: –0.42, SD: 1.34
Wasting (WHZ ≤ –2)	13.4% (18/134)
Nutritional status (>5 years)	(307/441)
WAZ^†^	Mean: –0.71, SD: 1.38
HAZ	Mean: –1.11, SD: 1.26
BAZ	Mean: –0.01, SD: 1.31

BAZ = body-mass-index-for-age Z-score; HAZ = height-for-age Z-score; WAZ = weight-for-age Z-score; WHZ = weight-for-height Z-score.

* Children who had been dewormed in the previous 3 months were excluded from participation.

^†^ Children older than 10 years (*n* = 84), were not included in this calculation.

### Prevalence of infection with STH.

The prevalence of *Ascaris* and *Trichuris* infections before albendazole treatment was 33.8% and 34.5%, respectively (Table 2). As the study visits progressed, there was a significant decrease in the prevalence of *Ascaris* infections, with a prevalence of 9.2% in the second visit, 12.2% in the third visit, and 9.6% in the fourth visit. In contrast, for *Trichuris*, the prevalence of infection remained relatively high at 29.3% in the second visit, showing a moderate reduction during subsequent visits with a prevalence of 14.3% in the third visit and 8.5% in the fourth visit after combined albendazole and ivermectin treatment. Hookworm was rare in this population and present in only 0.9%, and *Strongyloides* was identified in 0.5% of pretreatment samples. Coinfections with *Ascaris* and *Trichuris* were common, with 20.7% of participants having a dual infection with these two STH at baseline. Only 52.3% of participants did not have any STH upon pretreatment assessment.

**Table 2:** Prevalence of helminth infection pretreatment and at three subsequent follow-ups by direct observation

STH	Visit 1	Visit 2	Visit 3	Visit 4
	(day 1)	(day 20)	(day 90)	(day 130)
	% (*n/N*)	% (*n/N*)	% (*n/N*)	% (*n/N*)
*Trichuris trichiura*	34.5 (147/426)	29.5 (119/403)	14.3 (55/384)	8.5 (32/375)
*Ascaris lumbricoides*	33.8 (144/426)	9.2 (37/403)	12.2 (47/384)	9.6 (36/375)
Hookworm	0.9 (4/426)	0.5 (2/403)	0.8 (3/384)	0.3 (1/375)
*Strongyloides stercolaris*	0.5 (2/426)	0.3 (1/403)	0.7 (1/384)	0.0 (0/375)
*Ascaris*/*Trichuris* coinfection	20.7 (88/426)	4.5 (18/403)	5.2 (20/384)	2.1(8/375)
*Ascaris lumbricoides* only	13.1 (56/426)	4.7 (19/403)	7.0 (27/384)	7.7 (29/375)
*Trichuris trichiura* only	13.8 (59/426)	24.8 (100/403)	9.1 (35/384)	6.9 (26/375)
No STH	52.3 (223/426)	66.0 (266/403)	79.1 (302/382)	84 (315/375)

STH = soil-transmitted helminth.

### Intensity of infection with STH at baseline.

The majority of participants (51.4%) that were positive for *Ascaris lumbricoides* at pretreatment assessment had moderate intensity infections, whereas 41.7% of infections were light and only 6.9% of infections were categorized as heavy at baseline (Table 3). The majority of *Trichuris* infections were light (75.5%) with 22.4% of infections categorized as moderate and only 2.1% of infections as heavy. All hookworm infections documented (4, or 1.1% of baseline samples) were light.

**Table 3:** Intensity of *Ascaris lumbricoides*, *Trichuris trichiura*, and Hookworm sp. infection at baseline in enrolled children

Intensity of Infection	Visit 1 (day 1)	Visit 2 (day 20)	Visit 3 (day 90)	Visit 4 (day 130)
	% (*n/N*)	% (*n/N*)	% (*n/N*)	% (*n/N*)
*Ascaris lumbricoides* *				
None	66.2 (282/426)	90.8 (366/403)	87.8 (337/384)	90.4 (339/375)
Light	41.7 (60/144)	54.1 (20/37)	63.8 (30/47)	58.3 (21/36)
Moderate	51.4 (74/144)	35.1 (13/37)	31.9 (15/47)	41.7 (15/36)
Heavy	6.9 (10/144)	10.8 (4/37)	4.3 (2/47)	0 (0/36)
*Trichuris trichiura* ^†^				
None	65.5 (279/426)	70.5 (284/403)	85.4 (328/384)	91.5 (343/375)
Light	75.5 (111/147)	87.4 (104/119)	89.3 (50/56)	93.8 (30/32)
Moderate	22.4 (33/147)	10.9 (13/119)	10.7 (6/56)	6.2 (2/32)
Heavy	2.1 (3/147)	1.7 (2/119)	0 (0/56)	0 (0/32)
Hookworms^‡^				
None	98.9 (422/426)	99.5 (401/403)	99.2 (381/384)	99.7 (374/375)
Light	100 (4/4)	100 (2/2)	100 (3/3)	100 (1/1)
Moderate	0.0 (0/4)	0.0 (0/2)	0.0 (0/0)	0.0 (0/0)
Heavy	0.0 (0/4)	0.0 (0/2)	0.0 (0/0)	0.0 (0/0)

* Light (1–4,999 egg/gram [epg]), moderate (5,000–49,999 epg), heavy (>50,000 epg).

^†^ Light (1–999 epg), moderate (1,000–9,999 epg), heavy (>10,000 epg).

^‡^ Light (1–1999 epg), moderate (2,000–3,999 epg), heavy (>4,000 epg).

### Follow-up and response to standard of care treatment.

**Figure 3.**
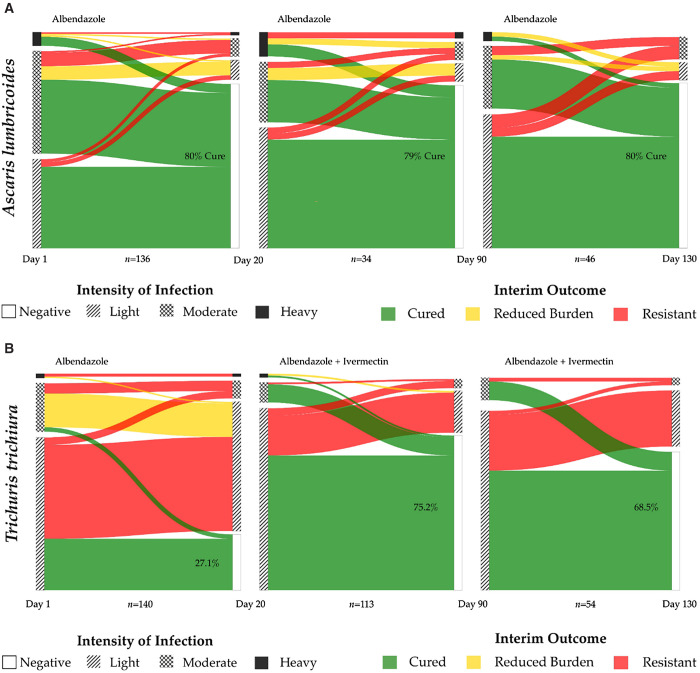
(**A**) *Ascaris lumbricoides* and cure rates at baseline with albendazole alone and then with combination treatment as defined by Kato–Katz. Flow diagrams showing outcomes for participants with stool samples positive for *Ascaris lumbricoides* at days 1, 20, and 90, grouped by WHO burden category and colored by outcomes after albendazole monotherapy as assessed at follow-up stool testing on day 20. On days 90 and 130, individuals received treatment as defined per stool testing results and either received albendazole 400 mg alone or in combination with ivermectin. Reduced burden is defined here as downgrade of infection intensity by at least one WHO burden category. Cure rates are noncumulative and based on the number of infected participants at the beginning of each time period. Each round of therapy resulted in a cure rate of 80% (first period), 79% (second period), and 80% (third period). The infection status of the population under study grouped by burden of infection for *Ascaris* during three periods: 1) enrollment and day 20 after albendazole therapy, 2) days 20–90, and 3) days 90–130 after their initial deworming. Each round of therapy resulted in a cure rate of 80% (first period), 79% (second period), and 80% (third period). Cure was attained independently of the burden level at the time of treatment. (**B**) Treatment outcomes for *Trichuris* infection after albendazole monotherapy and after combined therapy with albendazole and ivermectin. Flow diagrams showing outcomes for participants with stools samples positive for *Trichuris trichiura* at days 1, 20, and 90, grouped by WHO burden category and colored by outcomes after albendazole monotherapy at day 20, and after combined albendazole with ivermectin at days 90 and 130, respectively. Reduced burden is defined here as downgrade of infection intensity by at least one WHO burden category. Cure rates are noncumulative and based on the number of infected participants at the beginning of each time period. The infection status of the population under study grouped by burden of infection for *Trichuris* during 3 periods: 1) enrollment and day 20 after albendazole therapy; 2) days 20–90, and 3) days 90–130 after their initial deworming. As shown in the initial temporal window (baseline through day 20), the majority of children (72.9%) infected with *Trichuris* infection did not experience complete cure and continued to have the infection 20 days after being administered therapy under observation. Most children with *Trichuris* who were cured were in the light infection category. Children who were identified as persistently infected at day 20 received albendazole and ivermectin, which resulted in a cure rate of 75.2%, with nearly all cases of heavy and moderate infection attaining cure at day 90. Children who were infected with *Trichuris* at 90 days after the initial deworming period were again treated with single-dose albendazole and ivermectin, with a 68.5% cure rate.

Follow-up after programmatic treatment with a single dose of albendazole of 400 mg was performed (Supplemental Tables 1 and 2; Figure 3). The CR (C20) for *Ascaris* infections was 80.1% (95% CI: 73.5–86.8), and the ERR was 70.8% (95% CI: 57.8–88.7; Supplemental Table 2; Figure 3A). Before treatment, 6.9% of *Ascaris* infections were heavy, 51.4% were moderate, and 41.7% were light. After albendazole therapy, 10.8% were heavy, 35.1% were moderate, and 63.8% were light. For *Trichuris* infections, the CR at day 20 (C20) was only 27.1% (95% CI: 20.0–34.3) with an ERR of 29.8.% (95% CI: –1.40 to 57.5; Supplemental Table 2; Figure 3B). After treatment with albendazole alone, the number of heavy infections decreased from 2.1% to 1.7%, the number of moderate infections decreased from 22.4% to 10.9%, and the number of light infections increased from 75.5% to 87.4%. Treatment responses based on initial infection burden of *Ascaris* and *Trichuris* at the three treatment opportunities (D20, D90, D130) can be seen in Figure 3A and B.

### Response to albendazole and ivermectin single-dose combined therapy.

Treatment responses in children with *Trichuris* infection who received albendazole and ivermectin resulted in CRs of 75.2% (95% CI: 67.3–83.2%) and ERRs of 84.2% (95% CI: 61.3–93.8%) on the first round on day 90 and a CR of 68.5% (95% CI: 55.6–81.5%) and an ERR of 80.9 (95% CI: 59.3–93.6%) at day 130 (Figure 3B). Both the ERR and CR were approximately 3 times higher with dual therapy compared monotherapy at the both the 90- and 130-day time points (*P* <0.01 for both CR and ERR). All heavy *Trichuris* infections were cured or downgraded with dual therapy (Figure 3B).

A group of children that had infection with both *Ascaris* and *Trichuris* infections received this dual therapy, whereas those infected with *Ascaris* only or *Ascaris* and hookworm received albendazole alone. Cure rates for children infected with *Ascaris* infection alone treated on day 90 with albendazole was 83.3% (95% CI: 66.7–100) compared with 75.0% (95% CI: 50.0–93.75) for those treated with both albendazole and ivermectin. Egg reduction rates for *Ascaris* infection among children receiving dual therapy with albendazole and ivermectin were 67.4% (95% CI: 28.0–99.1) compared with 80.6% (95% CI: 46.6–100) for children with albendazole alone. Neither CRs or reduction in intensity of infection for ascariasis was significantly different between children treated with albendazole alone or albendazole combined with ivermectin at day 90 or 130.

### Reinfection.

Reinfection with *Ascaris* occurred at a rate of 18.6% after albendazole-only therapy between days 20 and 90, and 11.8% of those cured at day 90 were reinfected by day 130 after initial therapy (Table 4). Reinfection with *Trichuris* occurred in 41.2% of 34 children obtaining cure after single-dose albendazole between days 20 and 90 post-treatment. After dual therapy with albendazole and ivermectin, reinfections with *Trichuris* occurred in 7.1% of the 70 children cured between days 90 and 130 after initial deworming.

**Table 4:** Prevalence of reinfection according to the last visits

	*Ascaris lumbricoides*			*Trichuris trichiura*		
Visit	Cured*	Cases Reinfected^†^	Rate of Reinfection (%)	Cured	Cases Reinfected	Rate of Reinfection (%)
Pretreatment to 90-day visit	102	19	18.6	34	14	41.2
20 to 120 days after initial treatment	17	2	11.8	70	5	7.1

This subgroup analysis was limited to participants who were positive for *Ascaris* or *Trichuris* in the initial specimen. Cure rates are defined as the ratio of participants testing negative for the parasite after testing positive in the previous specimen, divided by the total number of participants testing positive in the prior specimen. In this group, specific treatment in accordance with WHO guidelines resulted in lower prevalence of infection at visits 3 and 4 for both *Trichuris* and *Ascaris*, suggesting that the plateauing prevalence of *Ascaris* infections observed for visits 2, 3, and 4 in the total study population reflects the contributions of new *Ascaris* infections. Follow-up treatment of participants who were positive for *Trichuris* with or without other soil-transmitted helminth coinfection at visit 2 with high-dose ivermectin in addition to albendazole was associated with lower egg counts for persistent infections with *Trichuris*, but not for *Ascaris*.

* After albendazole treatment of all study participants based on Ministry of Health guidelines.

^†^ After specific treatment by WHO guidelines for parasites identified at the second visit.

## DISCUSSION

The burden of STH infection was high in Iquitos, Peru in a community that had excellent access to intervention at the interval specified by the WHO. Five years after programmatic initiation, STH prevalence still approached 50% among children aged 2–11 years, and more than 50% of these infections were of at least moderate intensity. This high prevalence is unexpected because 67.9% of children had been dewormed in the previous 6 months, a number that is nearly equal to the population target of 75% specified by current guidelines that is predicted to diminish both disease burden and transmission. These findings contrast sharply with outcomes reported elsewhere. In Zimbabwe,^24^ helminth infections were nearly eliminated with the implementation of the same program in the same manner, albeit in a setting of lower prevalence and intensity. This suggests that in the Peruvian Amazon, and likely in other settings, intensification of anthelminthic treatment and or modification of the treatment used will be needed to attain 2030 goals to diminish the prevalence of moderate to heavy infections to <1%,^25^^,^^26^ as outlined by the WHO.^25^ A recent review of 15 countries reports a decrease in prevalence of STH infections of two-thirds,^27^ results that are clearly not manifest in Peru 6 years after program initiation of preventive therapy for STHs.

Our prospective observational study of single-dose albendazole documented lower CRs and ERRs for both ascariasis and trichuriasis than reference efficacy for albendazole. The WHO lists ERRs of 95% as reference efficacy for *Ascaris* infections and 50% for *Trichuris* infection based on the work of Vercruysse,^28^ compared with our observed ERR of 70.8% (95% CI: 57.8–88.7) for *Ascaris* infections and 29.8% (95% CI: 20.0–34.3) for *Trichuris* infections.^22^ The rates of albendazole efficacy are considerably lower than those measured in multiple recent studies^29^ and provide empiric evidence of diminished clinical efficacy of albendazole for the treatment of *Ascaris* infection in the region. Furthermore, *Trichuris* infection continues to respond poorly to albendazole therapy, an observation that is not new but has not yet driven a change in STH program policy in many settings, including Peru. There is a paucity of prior published studies in Peru documenting program efficacy of the current STH program. In Iquitos, a similar treatment efficacy study was performed in 2010 among fifth-grade children (approximately 10 years of age).^30^ Parasite prevalence was higher in this previous study compared with the current study, particularly that of hookworm, which is likely in part due to the differing age structure of the two study populations because hookworm prevalence generally peaks later in childhood than *Ascaris* or *Trichuris* infection. Efficacy of single-dose albendazole against *Ascaris* infection was 99.6% (95% CI: 97.8–100), and the ERR was 99.8 (95% CI: 99.3–100), notably higher than we have documented in the present study. Similar between the two studies was a CR of only 12.8% (95% CI: 8.0–18.2%) for *Trichuris* infection after single-dose albendazole in the prior study and 27.1% in the current study. These studies were separated in time by 10 years and further distinguished by 6 years of the consistent and the regular application of population-level treatment of 2- to 11-year-old children in the region. Although it is possible that other factors such as undernutrition and interactions with food may play a role in these differences, both these studies were conducted in the same area during programmatic application, and we do not feel these theoretical factors contributed to the disparate findings. The observed difference points to the need for periodic monitoring of treatment efficacy as part of programmatic monitoring and evaluation of STH control as opposed to exclusively measuring treatment coverage, a common practice in the monitoring of these programs.

Monotherapy for the control of STH has advantages, primarily those of simplicity and decreased logistic burden, which are notable advantages enabling both patient compliance and mass drug administration. Combination chemotherapy has the advantage of controlling the emergence of resistance and the ability to treat all four major STH (*Ascaris*, *Trichuris*, Hookworm, and *Strongyloides*) more effectively. The addition of ivermectin as a second agent added to benzimidazoles (albendazole) was recommended by the Global Program to Eliminate Lymphatic Filariasis for the control of lymphatic filariasis in areas where loiasis is not coendemic; this provides important data on safety and tolerance.^31^ The efficacy and safety have been independently evaluated in STH treatment,^29^ where it has been shown to be more efficacious in controlling *Trichuris* infection than albendazole alone.^32^ The dose used by clinicians in Peru was influenced by dose-finding studies demonstrating that doses of 600 *µ*g/kg are more effective than lower doses, particularly the standard dose of 200 mg/kg,^33^ and a single combined dose of each albendazole and high-dose ivermectin was recently noted to be well tolerated and highly efficacious in Honduras, with a CR of 88.6%.^16^ Alternative combinations with oxantel and albendazole have been highly efficacious but only slightly more effective than low-dose ivermectin and albendazole. Oxantel would not be expected to have the additional benefits that ivermectin has of treating *Strongyloides* and scabies and is only produced for human use in a limited number of countries in a combined form with pyrantel pamoate.

In our study, for children who were infected with *Trichuris* and remained persistently positive at 20 days after treatment with albendazole received combined single-dose therapy with albendazole (400 mg) and ivermectin (600 *µ*g/kg), the CR increased to 75%, and all heavy infections were either cured or downgraded. Despite the increased logistic complexity of an antihelminthic strategy that contains more than one agent, this alternative regimen retains the principal feature of logistic feasibility for mass drug administration—the application of complete therapy in a single dose. Note should be made of trend in decreased efficacy of dual therapy of albendazole and ivermectin in the treatment of *Ascaris* infection, but this difference was not statistically significant with broadly overlapping confidence intervals due to a relatively small sample size.

Between the first and third samples, the prevalence of reinfection was 18.6% for *Ascaris lumbricoides*, whereas between the first and third samples, the prevalence of reinfection for *Trichuris trichiura* was 41.2%. Between the third and fourth samples, the prevalence of reinfection was 11.8% for *Ascaris lumbricoides* and 7.1% for *Trichuris trichiura*, indicating intense transmission in this area and suggesting a need for increased treatment frequency, more efficacious antihelminthics, or combined strategies to decrease the intensity of infection in this area with concurrent interventions to improve water and sanitation infrastructure. Although single stool exams may mis-categorize individuals as being reinfected (by failure to detect a persistent low-burden infection at a single time point), we feel that the failure of multiple rounds of therapy seen in this study is of clinical and public health importance. Studies employing parasite genotyping would be required to distinguish definitively between persistent positivity with intermittent detection and reinfection.

The principal limitation of this study is that the comparison of treatments was not done after randomization, and treatment of previously treated children, who are likely to have had less intense infections may increase the apparent efficacy of second-round therapies. Randomized clinical trials would add additional strength to the evidence of the relative efficacy of albendazole versus albendazole and high-dose ivermectin and possibly alternative combined therapies^34^^–^^37^ to increase the efficacy of STH control programs in the area.

## CONCLUSION

The results of the study indicate that the albendazole has decreased efficacy against both *Ascaris* and *Trichuris* infection in the Peruvian Amazon. Alternative strategies that use different agents or combined therapy are needed to decrease rates of transmission and burden of STH in preschool and school-age children.

## Supplemental Materials

10.4269/ajtmh.23-0497 Supplemental Materials
