# Supplementary material for: Efficacy of Single-Dose Albendazole and Albendazole Plus Ivermectin for Soil-Transmitted Helminth Infection in Children in the Peruvian Amazon
Source: Am J Trop Med Hyg. 2024 May 28;111(1):80–8. doi: 10.4269/ajtmh.23-0497 (PMC11229650; doi:10.4269/ajtmh.23-0497)
Supplement: Supplemental Materials [file tpmd230497.SD1.pdf]

Supplementary Table 1

Ascaris lumbricoides and *Trichuris trichiura* positivity by follow-up visit

	Follow-Up Visit			
	Visit 1* (baseline)	Visit 2 @ (day 20)	Visit 3 & (day 90)	Visit 4 & (day 130)
Number of participants	426	403	384	375
Ascaris lumbricoides				
Positive	144 (33.8%)	37 (9.2%)	47 (12.2%)	36 (9.4%)
EPG Arithmetic mean	14752.67	17249.51	12193.28	8168.33
(SD)	19403.82	30462.41	25479.52	11593.38
Trichuris trichiura				
Positive	147 (34.5%)	119 (29.5%)	56 (14.6%)	32 (8.5%)
EPG Arithmetic mean	1093.796	972	452.3571	203.25
(SD)	2372.11	3106.934	755.71	302.83

* Before receiving treatment; SD = Standard Deviation

@ Following albendazole treatment as per Peruvian Ministry of Health guidelines

& Following specific treatment by WHO guidelines for parasites identified in at the previous visit

Supplementary Table 2

Ascaris lumbricoides and *Trichuris trichiura* Cure rate and Egg Reduction Rate (EGG) by to follow-up visit

Ascaris lumbricoides	Albendazole			Albendazole + Ivermectin		
	N +	%ERR (95% CI)	Cure Rate (95% CI)	N+	%ERR (95% CI)	Cure Rate (95% CI)
Day 0	144	NA	NA		NA	NA
Day 20	18	70.8 (57.8 - 88.7)	80.1 (73.5 - 86.8)	16	NA	NA
Day 90	26	80.6 (46.7 - 100)	83.3 (66.7 - 100)	20	67.4 (28.0 - 99.1)	75.0 (50.0 - 93.8)
Day 130	5	73.8 (-11.9 - 98.5)	80.8 (65.4 - 96.2)	4	75.8 (10.5 - 99.9)	80.0 (60.0 - 95.0)

Trichuris trichiura	Albendazole			Albendazole + Ivermectin		
	N +	%ERR (95% CI)	Cure Rate (95% CI)	N+	%ERR (95% CI)	Cure Rate (95% CI)
Day 0	140	NA	NA		NA	NA
Day 20	102	29.8 (-1.40 - 57.5)	27.1 (20.0 - 34.3)	113	NA	NA
Day 90		NA	NA	54	84.2 (61.3 - 93.8)	75.2 (67.3 - 83.2)
Day 130		NA	NA	17	80.9 (59.3 - 93.6)	68.5 (55.6 - 81.5)
